# An Immunohistochemical Study of Proliferation of Human Fetal Heart Cardiomyocyte With Phospho-Histone H3 Antibody

**DOI:** 10.7759/cureus.41159

**Published:** 2023-06-29

**Authors:** Arundhati Kar, Mukund Sable, Anbarasan A, Saubhagya K Jena, Prabhas R Tripathy, Manisha Gaikwad

**Affiliations:** 1 Anatomy, Institute of Medical Sciences (IMS) and SUM Hospital, Bhubaneswar, IND; 2 Pathology and Laboratory Medicine, All India Institute of Medical Sciences, Bhubaneswar, Bhubaneswar, IND; 3 Cytogenetics, Christian Medical College (CMC) Vellore, Vellore, IND; 4 Obstetrics and Gyenacology, All India Institute of Medical Sciences, Bhubaneswar, Bhubaneswar, IND; 5 Anatomy, All India Institute of Medical Sciences, Bhubaneswar, Bhubaneswar, IND

**Keywords:** fetal, phh3 index, cardiomyocyte, proliferation, human

## Abstract

The rapid proliferation of cardiomyocytes in mammals occurs during fetal life. But in postnatal life, this capacity of proliferation is reduced or lost as they exit the cell cycle. However, the cardiomyocytes don't show the same activity for different species. In human fetuses or in adult life, the capacity of the proliferation of cardiomyocytes and their response to an injury are not understood yet. In this study, we have done an immunohistochemical study using phospho-histone H3 (PHH3) to observe human fetal cardiomyocytes' proliferative activity. The heart specimens from the fetal autopsy of spontaneously aborted and stillborn human fetuses were subjected to immunohistochemical study using PHH3 antibody, and comparison between the PHH3 index (number of PHH3 positive cells per 1000 number of cardiomyocytes/high power field [HPF]) of myocardial regions was done using appropriate statistical tests. A total of 17 fetal hearts were included in our study. In the left ventricle, right ventricle, right atrium, and interventricular septum, the PHH3 index of myocardium was significantly higher over the pericardial region (p-value 0.002, p-value <0.001, <0.001, and 0.009 respectively) as compared to the region of over the endocardium and the middle part of the myocardium. The PHH3 index of the pericardial region of the left ventricle was significantly correlated with the maximum thickness of the left ventricle.

## Introduction

In mammals, cardiomyocytes proliferate rapidly during fetal life, but they lose the capacity to exit the cell cycle in immediate postnatal life. Species-specific differences among the activity of cardiomyocytes also exist. So, it is difficult to assume the exact sequence of actions in human fetal cardiomyocytes. The proliferative capacity of cardiomyocytes and their response to an injury in adult life are not understood yet. In adult life, two cellular mechanisms can explain myocardial growth, i.e., cardiomyocyte enlargement and proliferation [1]. But predominantly, the growth of adult human myocardium is due to the increase in cardiomyocyte size (hypertrophy). However, cardiomyocyte hypertrophy does not allow the heart to restore its function after any significant injury [2].

A literature review shows that some cardiomyocyte cell cycle activity contributes to postnatal cardiomyocyte proliferation [1]. This information gives an insight into the repair or regeneration of adult myocardium to maintain cardiac output in the compensated heart like in post-myocardial infarction. Knowledge about the mechanism that drives cardiomyocyte proliferation and differentiation can improve the treatment modality of different cardiac pathologies, ranging from cardiomyopathies, and heart failure to myocardial infarction [3].

Congenital heart disease (CHD) accounts for the most common congenital disabilities. CHDs like hypoplastic left heart syndrome (HLHS) and related single ventricle pathology develop cardiac dysfunction due to an increased ventricular workload [4]. Though, there have been recent advances in medical care, catheter interventions, and surgical procedures. A growing population of children and adults with CHD mandates new treatment strategies to optimize long-term outcomes [5].

In this study, we have used phospho-histone H3 (PHH3) as the primary antibody, to observe human cardiomyocytes' proliferative activity using an immunohistochemical study. Histone H3 phosphorylation at Threonine 3 residue (pT3) (specific for mammalian cells) is closely linked to chromosomal condensation; so, the monoclonal antibody directed to PHH3 has been proposed to detect mitotic cells. PHH3 is a mitosis-specific nuclear antibody, which was already proven to facilitate many tumors' mitotic count. It is a better mitotic marker as it increases the specificity of the quantification, as seen in the previous studies in different tissues, including cardiomyocytes of mice and rats [6]. It has also been used as a marker of proliferation in adult human cardiomyocytes [7]. Therefore, the aim of this study was to assess the proliferation of human fetal cardiomyocytes by using PHH3 immunohistochemistry.

## Materials and methods

The heart specimens were collected from the fetal autopsy of spontaneously aborted and stillborn human fetuses after ethical approval from the Institutional Ethics Committee (IEC) of All India Institute of Medical Sciences (AIIMS), Bhubaneswar (IEC/AIIMS BBSR/PG Thesis/2018-19/16). Visibly damaged hearts and fetuses with a maternal history of diabetes mellitus, chemotherapy, or known viral infections were excluded from our study. 

**Figure 1:**
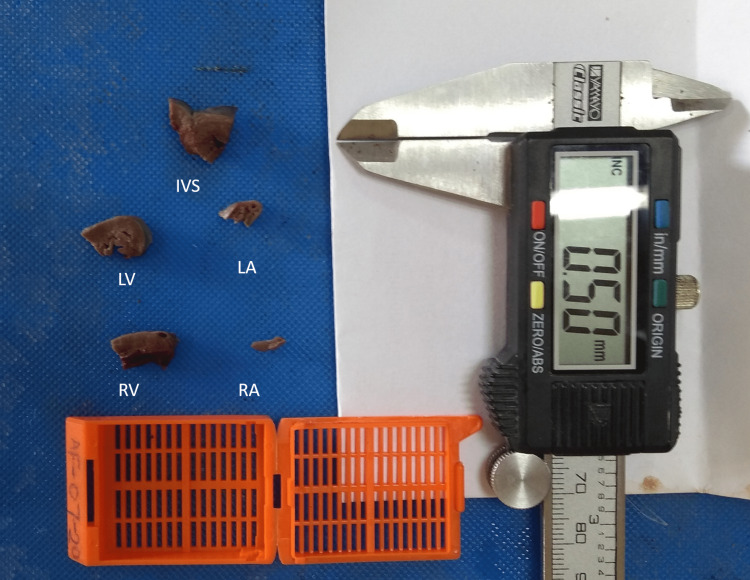
The measurement of maximum thickness using Vernier caliper and further with ImageJ software LV: left ventricle, RV: right ventricle, IVS: interventricular septum, LA: left atrium, RA: right atrium

Fetal hearts were collected during the autopsy. Weight (W) and the gestational age of fetuses were documented. Other measurements of the heart, including perimeter (P) of the heart at the level of atrio-ventricular septum (AVS), maximum anteroposterior (AP) diameter on the left and right side, maximum transverse (T) diameter at AV sulcus were done by using a flexible measuring tape. Tissue sections were taken from the wall of all four chambers and the interventricular septum (IVS), including the septum at the region of maximum thickness [8,9]. The thickness of the wall of all four chambers and IVS was measured using a digital vernier caliper (Figure 1).

Measurement of thickness was done using ImageJ software from the images taken by using a slide caliper (sensitivity- 0.02mm, least count- 0.1mm, make-up hardened stainless steel) as the standard of measurement [10].

The tissues are fixed in 10% neutral-buffered formalin for two to four days with an identifying label on cassettes. Then the tissue blocks were processed by the automated tissue processing method and embedded in paraffin blocks. Tissue sections of size approximately four μ thickness were taken in the form of a ribbon. The ribbon was gently placed on the surface of the water in a preheated water bath whose temperature was set at 50-60 ^0^C in order to remove folds of ribbon. The sections were lifted from the water bath and placed onto adhesive slides. Haematoxylin and eosin (H&E) staining was done on the tissues to see if the architecture of the tissues is preserved or not. The tissue sections showing preserved cellular architecture were acquired again and dried on a plate at 45-50 ^0^C overnight in an incubator for immunohistochemistry (IHC).

For the immunohistochemical study, the paraffin-embedded tissue sections were taken on 3-aminopropyl tri ethoxy silane (APES) coated slides. They were deparaffinized using xylene and ethanol. Sections were rehydrated by using an increasing percentage of alcohol. The antigen retrieval was then performed using heat-induced epitope retrieval in a citrate buffer (pH=6.1). After cooling the slides, endogenous peroxidase activity of the tissue section was blocked by incubation with a peroxidase blocker from En Vision Kit (Dako, Hamburg, Germany). Then slides were incubated with rabbit monoclonal anti-PHH3 antibody (PathnSitu, Livermore, CA, USA) ready to use (RTU) for 45 minutes. This is the primary antibody. The bound primary antibody was detected by using a secondary antibody with the En Vision TM detection system (Dako). For this method, 3,3 diaminobenzidine (DAB) was used as chromogen in a dark room setting, and mild counterstaining was performed with hematoxylin. Positive control slides (from a reactive lymph node) were included with each staining session. For negative control, the primary antibody was skipped with the rest steps intact.

**Figure 2:**
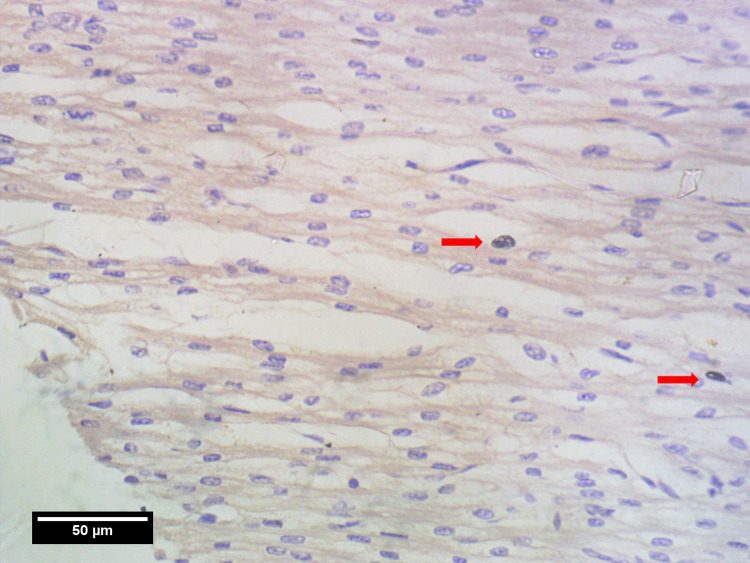
High power field view in eM region showing two PHH3-positive cells (red arrows) eM: myocardium towards endocardium, PHH3: phospho-histone H3

**Figure 3:**
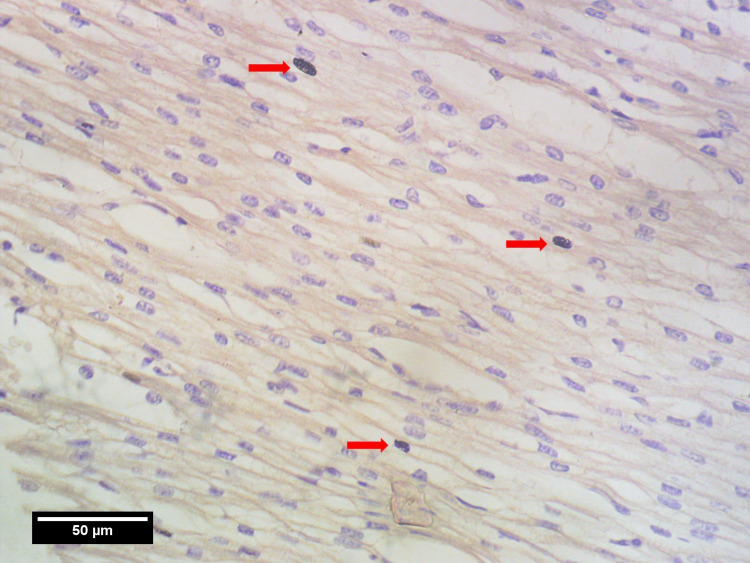
High power field view in mM region showing three PHH3 positive cells (red arrows) mM: middle part of the myocardium, PHH3: phospho-histone H3

**Figure 4:**
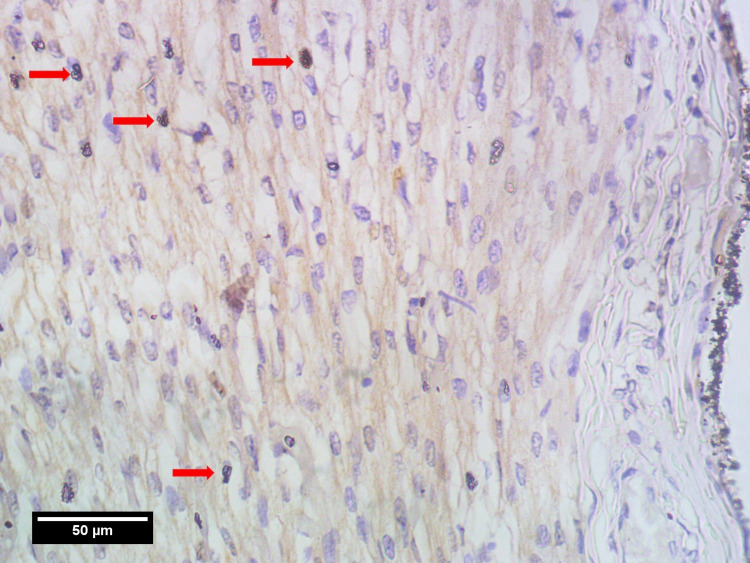
High power field view in pM region showing four PHH3 positive cells (red arrows) pM: myocardium toward pericardium, PHH3: phospho-histone H3

Photomicrographs were taken using a light microscope (Magnus; Olympus Opto Systems, Noida, India) at 40x magnification and Amscope 5 Mpx (Irvine, CA, USA) camera and were analyzed. The whole thickness of the myocardium in the left ventricle (LV), right ventricle (RV), and IVS were separated into three areas by two random imaginary lines. The three regions are: towards the epicardium (pM), towards the endocardium (eM), and the region in between them (mM). Nine photomicrographs were taken per tissue section with three photos from each region (i.e. pM, mM, eM) in high power field (HPF) (×40) [11-13]. For both atriums, the myocardium was separated into two regions i.e. pM&eM instead of three regions. The images were taken in the area of the highest thickness of each chamber, assuming maximum mitotic activity. All the photomicrographs (Figures 2, 3, 4) were observed for mitotic activities i.e. PHH3 index using FUJI 2.4 software. The maximal mitotic count was obtained by summing up the number of PHH3-positive nuclei counted on immunohistochemically stained sections in nine consecutive fields, at three separate regions. PHH3 index (number of PHH3 positive cells per 1000 number of cardiomyocytes/HPF) was calculated for the three regions of the myocardium.

In each field, the total number of cells was counted by counting the nuclei and analyzed by using Fiji/ImageJ free software from NIH (downloaded from http//imagej.nih.gov/). The calculation of the cells by using the HPF images of IHC slides. DAB-positive cells were counted manually.

Continuous variables were expressed as mean ± standard deviation and categorical variables were expressed as frequency and percentage. The relationships between gestational age, weight, and PHH3 index were evaluated using the Pearson correlation test. The Shapiro-Wilk test tested the normality of variables. Comparison of parametric variables between groups was done by ANOVA test. The Mann-Whitney U test was used for the comparison of nonparametric continuous variables between two groups, and the Kruskal-Wallis test was used for comparison between more than two groups. All statistical analysis was done by using SPSS version 21 (SPSS Statistics for Windows, Version 21.0; IBM Corp., Armonk, NY, USA). A p-value of less than 0.05 was taken as significant.

## Results

A total of 49 fetuses were selected for our study. Depending upon the tissue preservation assessed on H&E staining, 17 fetuses were included in this study. Thirty-two cases, with loss of tissue architecture or changes of autolysis, were excluded from the study. Out of 17 fetuses, nine (53%) were female and eight (47%) were male fetuses. The age of fetuses extends from 12 weeks to 40 weeks five days. Out of these fetuses, one (5.9%) was from the first trimester (0-12th weeks), seven (41.2%) were from the second trimester (12th-28th weeks), and nine (52.9%) were from the third trimester (28th weeks-birth).

A comparison of the proliferative index of cardiomyocytes among each region (eM, mM, and pM) was done by using appropriate statistical methods and is shown in Table 1. In the left ventricle, the PHH3 index was significantly higher over the pericardial region (p-value 0.002) as compared to the other two regions. There was no significant difference between the two regions in the left atrium (p-value 0.128). Similarly, the PHH3 index of myocardium towards the pericardium of the right ventricle, right atrium, and interventricular septum was significantly higher compared to other regions (p-value <0.001, <0.001, and 0.009 respectively).

**Table 1:** Comparisons of PHH3 index among the three regions of the myocardium of LV, RV, IVS, LA, RA LV: left ventricle, RV: right ventricle, IVS: interventricular septum, LA: left atrium, RA: right atrium, eM: myocardium towards endocardium, mM: middle part of the myocardium, pM: myocardium toward pericardium, PHH3: phospho-histone H3 @- p-value calculated using Kruskal- Wallis test,  #- p-value calculated using Mann-Whitney U test *- in post hoc analysis, the PHH3 index of pM is significantly different from that of eM and mM, but no difference between the index of mM and eM

Areas	Regions			
	eM	mM	pM	P Value
LV	7.386 ± 2.800	7.498 ± 1.995	8.612 ± 1.414	0.002@*
RV	7.568 ± 2.505	8.141 ± 2.830	9.376 ± 2.053	<0.001@*
IVS	8.843 ± 2.494	9.247 ± 2.520	10.649 ± 2.480	0.009@*
LA	5.732 ± 1.691		5.959 ± 1.814	0.128@
RA	7.386 ± 2.800		8.612 ± 1.414	0.002@*

The Spearman correlation of the PHH3 index of different regions of the myocardium of chambers with the maximum thickness of respective chambers was evaluated. (Table 2). The PHH3 index of the pericardial region of the left ventricle was found to have a significant positive correlation with the maximum thickness of the left ventricle. But the PHH3 index of the myocardium of other chambers had no significant correlation with the wall thickness of respective chambers.

**Table 2:** The correlation of PHH3 index of different regions of the myocardium of chambers with the maximum thickness of respective chambers LV: left ventricle, RV: right ventricle, IVS: interventricular septum, LA: left atrium, RA: right atrium, eM: myocardium towards endocardium, mM: middle part of the myocardium, pM: myocardium toward pericardium, PHH3: phospho-histone H3

	PHH3 Index	Correlation coefficient	p-value
LV	eM	0.407	0.105
	mM	0.438	0.078
	pM	0.553	0.021
RV	eM	-0.328	0.198
	mM	-0.285	0.267
	pM	-0.306	0.233
LA	eM	0.478	0.099
	pM	0.237	0.457
RA	eM	0.228	0.379
	pM	0.452	0.068
IVS	eM	-0.48	0.051
	mM	-0.333	0.192
	pM	-0.469	0.057

The mean PHH3 index of the three regions of the myocardium during each trimester was compared and shown in Table 3. There was no significant difference in the PHH3 index of the three myocardial regions of the left ventricle during different trimesters. But in the right ventricle, the mean PHH3 index of the second trimester was significantly higher than the PHH3 index of the first and third trimesters over all three myocardial regions. Over the atria, there was no significant difference between trimesters. For the interventricular septum, the PHH3 index of the second trimester of endocardial and pericardial myocardium was significantly higher compared to the first and third trimesters.

**Table 3:** Comparison of PHH3 index of the three regions of the myocardium during each trimester LV: left ventricle, RV: right ventricle, IVS: interventricular septum, LA: left atrium, RA: right atrium, eM: myocardium towards endocardium, mM: middle part of the myocardium, pM: myocardium toward pericardium, PHH3: phospho-histone H3

		First (N=1)	Second (N=7)	Third (N=9)	P value
LV	eM	5.911	6.426 ± 1.921	8.297 ± 3.301	0.279
	mM	6.446	6.649 ± 1.043	8.275 ± 2.387	0.057
	pM	8.16	7.889 ± 1.470	9.226 ± 1.214	0.124
RV	eM	6.77	9.133 ± 3.142	6.440 ± 1.220	0.006
	mM	7.136	9.902 ± 3.871	6.883 ± 0.404	0.003
	pM	8.249	10.779 ± 2.639	8.410 ± 0.522	0.007
LA	eM	5.348	6.120 ± 0.253	6.302 ± 0.334	0.15
	pM	6.329	6.490 ± 0.453	6.459 ± 0.289	0.91
RA	eM	5.245	6.055 ± 0.413	6.477 ± 0.442	0.062
	pM	5.695	6.922 ± 0.181	7.082 ± 0.402	0.199
IVS	eM	7.519	10.751 ± 2.555	7.506 ± 1.458	0.01
	mM	8.178	10.704 ± 3.486	8.232 ± 0.653	0.061
	pM	9.25	12.402 ± 2.948	9.441 ± 1.097	0.014

## Discussion

Previously a number of studies had been conducted on fetal heart morphometry which can be divided into two types. Some studies had used echocardiography (USG) to assess the fetal heart, its anatomy, and the gradual development through trimesters [14]. Other studies were post-mortem studies conducted on aborted fetuses with an emphasis on morphometry and stereology. There had been very few studies conducted on the proliferation of cardiomyocytes in fetal hearts. Its dynamics according to various regions of myocardium and age was the novelty of this study.

For assessment of proliferation, markers like Ki-67 and mitotic activity index (MAI) were used earlier in various studies on cardiomyocytes. Recently, PHH3 was being used in malignancies like breast carcinoma and meningioma to assess proliferative activity instead of Ki-67 and has been proven to be comparable to and better than previous mitotic indicators [15,16].

PHH3 had also been used in some living animal models like mice, murine, etc, to see the response of proliferation towards factors like Wnt/Hippo Pathway, and Pitx2c [17,18]. The genetic effect (TbX20 gene and its cascade) on cardiomyocyte maturation and proliferation using PHH3 with differential results in embryonic versus the fetal heart of mouse has also been documented [19], but the regional variation of proliferation in the human fetal heart is yet to be established.

In the present study, the fetal heart myocardium was divided into three regions, and the PHH3 index was calculated for each area of LV, RV, LA, RA, and IVS. Though PHH3 had been used as a marker of mitoses in various tissues, its use for the estimation of proliferative activity of human fetal cardiomyocytes was novel and had not been done earlier. We also compared the PHH3 index between the three areas of the myocardium (eM, mM, pM) of all chambers and IVS. The area of the myocardium adjacent to the epicardium (pM) was found to have a significantly higher PHH3 index compared to the other regions. This was suggestive of higher proliferative activity of the cardiomyocytes nearer to the pericardium.

The epicardial cells are an essential factor in the proliferation of cardiomyocytes. It is stimulated by the presence of factors like thyroid hormone, oxidative stress, and nutrition of the individual and by their interaction [20,21]. There were two mechanisms hypothesized which can explain our finding. The first one is the epicardial-derived cardiomyocytes. Various mouse model studies for lineage tracing using Scleraxis, WT1, and TBX18 to trace cells have demonstrated epicardial-derived functionally active cardiomyocytes [22]. The second mechanism is the epicardium acting as the paracrine organ that supports myocardial growth [22,23]. The factors secreted from this layer help in the exchange of signals between the myocardium and the epicardium. Due to this exchange, the proliferation and differentiation of myocardium occurs [24]. Other examples of epicardial-myocardial signaling are retinoic acid, and FGF-9, which are known contributors to cardiomyocyte proliferation [25,26]. Defective retinoic acid receptors or FGF-9 receptors (related to epicardium) are associated with a decreased cycling of cardiomyocytes [27].

This emphasizes the importance of the epicardium in cardiac development, by contributing cells as well as essential cytokines and growth factors to induce myocardial development. Another study by William et al. (2014) showed that the extracellular matrix (ECM) is the induction factor for cardiomyocyte proliferation [28]. In their study, fetal cardiomyocytes had significantly higher proliferation as evidenced by increased PHH3-positive cells after being exposed to five days of extracellular matrix. Our findings strengthen this important role of the epicardium in the proliferation of myocardium.

When the mitotic index was compared between the three trimesters, we found that the proliferative activity of cardiomyocytes increased towards the second trimester than the first trimester. But the proliferative activity of certain myocardial regions decreased towards the third trimester for RV and IVS and remains unchanged for all regions of the myocardium of LV. This decrease or unchanged proliferative activity of myocardium despite the increasing thickness of the ventricular as well as septal wall suggests increased hypertrophy of cardiomyocytes in the third trimester. Mandarim-de-Lacerda (1997) studied the stereology using the volume densities of the cardiac myocyte and interstitium, and the numerical density of the cardiac myocytes. They concluded that the development of the myocardium at the end of the fetal human period was mainly due to hypertrophy of the myocyte as well as interstitium [29].

The present study also includes the correlation between the thickness of chambers with the proliferative index of different regions of the myocardium but could not find a statistically significant correlation between the parameters except for a positive correlation between the thickness of the left ventricle and the PHH3 index of the left ventricular pericardial myocardium.

This study shows that the myocardium towards the epicardium has more cells undergoing mitosis in fetal life in LV, RV, and IVS. Again, mitosis is more in the second trimester in the right ventricle and interventricular septum. Hence cells of ventricular (preferably right ventricular) or septal cardiomyocytes as well as the adjacent epicardial cells can be used like stem cells, by isolating them [24,30].

The small sample size was one of the limitations of our study. Non-inclusion of the weight of the fetal heart and its correlation with the PHH3 index was also another limitation of our study, in the presence of which we could have compared the proliferative activity of cardiomyocytes during the course of development. We could acquire only one sample from the first trimester.

## Conclusions

This study establishes that PHH3 can be used as a marker for fetal cardiomyocyte proliferative activity. It also supports the earlier findings of pericardium being an inducer of cardiomyocyte proliferation and hypertrophy of cardiomyocytes being the major contributor to cardiac development in late fetal life.

This study proves that the second trimester can be the best time of fetal life during which the proliferating cells (in the right ventricular or septal wall with adjacent epicardial cells) can be used as stem cells after their isolation.
